# What makes public health-related networks work? Qualitative insights from the Eastern Mediterranean and Arab world

**DOI:** 10.3389/fpubh.2026.1880808

**Published:** 2026-08-03

**Authors:** Ahmed Mandil, Maya Hassan, Iman Nuwayhid

**Affiliations:** 1 Department of Epidemiology, High Institute of Public Health, University of Alexandria, Alexandria, Egypt; 2 Faculty of Health Sciences, American University of Beirut, Beirut, Lebanon

**Keywords:** Arab world, EMR, health, networks, public health, Eastern Mediterranean

## Abstract

**Introduction:**

There is limited research on public health-related networks in the Eastern Mediterranean Region (EMR) and Arab world, where vulnerabilities are multiple, interconnected and the need for effective collaborative action is urgent. This study aimed at gaining a deeper understanding of the experiences of current and former public health-related networks in the EMR/Arab world, identifying the factors that facilitated or hindered their functioning, and providing guidance on best practices that can inform the design of sustainable and resilient regional networks.

**Methods:**

We identified nine regional public health-related networks in the EMR/Arab world. A desk review of these networks was conducted followed by key informant interviews with 16 current or former members of these networks. Inductive thematic analysis was performed on the data generated from these semi-structured interviews.

**Results:**

Analysis showed that networks exist primarily to build connections among individuals and institutions in the region. Successful networks are marked by strong institutionalization of capacity-building initiatives and rapid mobilization into public health crises. However, networks often struggle or fail due to lack of sustained, flexible financing and fragmented governance, including dependence on individual leaders. Key recommendations from participants included institutionalizing leadership, diversifying financing, enhancing regular communication, strategic planning, and investing in new leaders. Informants widely agreed that robust, sustainable networks are vital to advancing public health in the region.

**Conclusion:**

To foster resilient and sustainable public health networks across the region, future initiatives should focus on demonstrating value, building trust, and strengthening institutional frameworks. Networks should learn from each other’s experiences. The literature on regional networks has mostly emphasized activities and achievements but offered limited critical analysis of underlying enabling and constraining conditions.

## 1 Introduction

Networks are relational organizational forms that interconnect individuals, groups, or organizations within a particular domain of knowledge and practice, interacting socially and exchanging knowledge to facilitate the accomplishment of a shared goal (1–3). Common goals include knowledge growth or co-creation (4–6), professional development (7), cooperative problem-solving, sustained energy, and attribution of values (8). Networks provide an alternative to managing through hierarchies and can achieve collaboration by creating opportunities for different players to recognize mutual benefits and ‘win–win’ situations (9). In the shifting landscape of public health, these benefits are particularly pressing; networks are not merely mechanisms of effectiveness, but constitute a key infrastructure on which public health responses succeed or fail (10).

The urgency of networks is amplified by the contemporary global public health landscape, which is loaded with multiple and concurrent crises and challenges, some of which presenting a threat to our very existence, be it climate change and environmental degradation, pandemics, aging and increase in non-communicable diseases, and geopolitical turmoil (11). Most of these challenges are unprecedented both in scale and complexity and stop at no national border (11). In this context, the need for health and non-health actors to collaborate has become as vital as the technical excellence of interventions they deliver (12, 13).

Public health networks may comprise *individuals*, such as professionals and specialists, with a shared set of commonalities or profession. By leveraging their members’ expertise, these networks promote public health, exchange new knowledge and discoveries, and represent their field (10, 14). Networks may also comprise *institutions*, including educational and professional bodies, that work together to share governance and educational or practice experiences, while addressing the growing challenges in improving health (10, 14). In addition, networks may include both *individuals* and *institutions*. Several examples demonstrate the value of such collaboration. For example, the Association of Schools of Public Health in Africa (ASPHA) has contributed to addressing key gaps in the COVID-19 response (15), while the Association of Schools of Public Health in the European Region (ASPHER) has played a role in developing a roadmap to professionalize the public health workforce in the European Region (16). These collaborations highlight that confronting health adversities is possible through strategic collaboration, unified commitment, and robust networking toward a shared purpose. Such efforts also align with the World Health Organization (WHO) mandate of strategic partnerships as a cornerstone in addressing the complexities of global, regional, and national public health challenges (17). However, coordinated efforts remain uneven across and within regions. Research on networks has tended to emphasize the business and enterprise sector rather than public health. The literature documents many one-off cases of success or failure in business and enterprise networks (18, 19). Relatively little has been published about public health networks, particularly “regional public health networks,” and even less about how they evolve, adapt, succeed or fail over time.

These gaps are especially concerning in the Arab world and the Eastern Mediterranean Region (EMR), where vulnerabilities are high and the need for cohesive public health action is most urgent. The EMR and the Arab world face a myriad of complex public health challenges resulting from armed conflicts and political instability, forced displacement, heterogeneous and inequitable opportunities for education, employment, or health care, re-emerging communicable diseases, a rising burden of non-communicable diseases, road and work-related injuries, climate change, and more (20). Recently, between 2023–2025, the region experienced a series of crises, including earthquakes in Afghanistan, Syria and Morocco, the wars in Gaza and Lebanon, multiple disease outbreaks, devastating floods in Libya, and protracted conflicts in Somalia and Sudan (21). Despite growing urgency, collaborative public health efforts in the region have been fragmented and often unsustainable.

Over the past decades, the EMR and the Arab world have witnessed the establishment of several public health networks, such as the Eastern Mediterranean Regional Academic Institutions Network (EMRAIN), the Reproductive Health Working Group (RHWG), the Eastern Mediterranean Public Health Network (EMPHNET), and others. These efforts provide a rich landscape of best practices, lessons learned, successes worth replicating, and pitfalls to avoid; yet there is a limited empirical understanding of what has worked, what has not, and why.

Such scarce documentation represents a crucial gap in both academic and policy literature, especially in the context of the EMR and the Arab world. Documenting and synthesizing these experiences represent a necessary step in shaping a more viable path forward. Thus, the goal of this study is to gain a deeper understanding of the experiences of current and former public health and related networks in the EMR and the Arab world, identify the factors that facilitated or hindered their functioning, and provide guidance on best practices that can inform the design of sustainable and resilient regional public health networks.

## 2 Methods

### 2.1 Study design

We conducted a qualitative study, including a desk-based review and key informant interviews (KIIs) with current or past members of current and former regional public health-related networks in the EMR and the Arab World, defined as networks addressing health issues beyond clinical care and encompassing related fields such as the social and behavioral sciences. The data from the desk-based review was used to enhance the understanding of the experiences of public health-related networks in the region. Whereas the data from the qualitative semi-structured interviews helped explore the experiences of networks’ members, with special emphasis on enablers and challenges (22, 23).

### 2.2 Setting

This study was implemented using online platforms. Participant recruitment and interviews were performed between August 2024 and February 2026.

### 2.3 Sampling and recruitment

The research team identified a set of regional public health networks operating in the EMR and the Arab world. Network identification was guided by several criteria, including variation in organizational structure (individual-based, institution-based, or hybrid), thematic focus within public health, and variation in stage of development and longevity, informed by the research team’s prior professional and research engagement with regional public health networks. This process yielded a list of nine networks that served as the sampling frame for the study, namely, the Arab Forum for Social Sciences and Health [AFSSH]; Arab Public Health Association [ArPHA]; Eastern Mediterranean Association of Medical Editors [EMAME]; Eastern Mediterranean Public Health Network [EMPHNET]; Eastern Mediterranean Regional Academic Institutions Network [EMRAIN]; Middle East and North Africa chapter of the International Epidemiological Association [IEA/MENA]; Network for Evidence and Data to Policy [NEDtP]; Reproductive Health Working Group [RHWG]; and the MENA-Health Policy Forum.

Purposive sampling was then used to recruit key informants who are or were members of such networks, or who have had substantial experience working with or within such networks. From each identified network, two to four potential participants were identified and invited to participate. Snowball sampling was also used, with initial participants recommending other relevant individuals to expand the pool. Although the Arab Council for the Social Sciences (ACSS) was not included in the original list of networks, several interviewees highlighted its relevance given its extensive experience in supporting research, capacity building, and knowledge production related to health from a population perspective. Accordingly, the research team broadened the scope of the study to include “public health–related networks,” defined as networks addressing health issues beyond clinical care and encompassing related fields such as the social and behavioral sciences.

Key informants were approached for participation via email, and recruitment continued until thematic saturation was reached (24, 25). In total, semi-structured interviews were conducted with sixteen individuals who consented to participate, several of whom reported experiences with more than one network. Despite repeated attempts, no key informants from one of the identified networks were available for interview. Participating key informants were affiliated with ACSS (*n* = 1), AFSSH (*n* = 4), ArPHA (*n* = 1), EMAME (*n* = 3), EMPHNET (*n* = 1), EMRAIN (*n* = 3), IEA/MENA (*n* = 3), NEDtP (*n* = 1), and RHWG (*n* = 3), while two individuals reflected on their broader experience with regional public health networks. The final sample size was based on the saturation principle, as no new information emerged after the sixteenth interview.

### 2.4 Data collection

#### 2.4.1 Desk-based review

The desk-based review focused specifically on documents related to the regional networks identified for the study. Two search methods were utilized: (1) electronic database searches, which included PubMed, Ovid Medline, Academic Search Premier, WHO Index Medicus for the Eastern Mediterranean Region (IMEMR), Google Scholar, EBSCO, and grey literature; and (2) a bibliographic review of reference lists from papers selected through these electronic searches. As an initial step, a subset of five articles was identified to inform the search strategy (see online Supplementary file 1). MeSH terms and keywords from these articles guided the design of search strategies. Documents concerning national or sub-national networks were excluded. In total, 53 documents were retrieved, including research articles, commentaries, viewpoints, reviews, editorials, keynotes, concept notes, and conference reports. The 53 documents were analyzed by network, type, and year of publication (Table 1). We used these data to provide background context and enhance the interpretation of the qualitative findings, rather than conducting a scoping review.

**Table 1:** Characteristics of the 53 documents included in the document review.

Characteristic	*n* (%)
Network represented	
The Eastern Mediterranean Public Health Network (EMPHNET)	14 (26.4)
Network of Institutions for Evidence and Data to Policy (NEDtP)	11 (20.8)
Arab Council for the Social Sciences (ACSS)	10 (18.9)
Reproductive Health Working Group (RHWG)	6 (11.3)
Eastern Mediterranean Association of Medical Editors (EMAME)	7 (13.2)
International Epidemiological Association (IEA)	2 (3.8)
The Arab Forum for Social Sciences and Health (AFSSH)	1 (1.9)
Arab Public Health Association (ArPHA)	1 (1.9)
The Eastern Mediterranean Region Academic Institutions Network (EMRAIN)	2 (3.8)
Document type	
Peer-reviewed journal articles	18 (34.0)
Organizational websites/about pages	8 (15.1)
WHO/organizational reports and meeting reports	11 (20.8)
Strategic documents, bylaws, and governance documents	7 (13.2)
Conference proceedings/workshop reports	6 (11.3)
Policy briefs, tools, and guidance documents	3 (5.7)
Publication year	
Before 2010	4 (7.5)
2010–2014	12 (22.6)
2015–2019	14 (26.4)
2020–2022	11 (20.8)
2023–2026	12 (22.6)
Source type	
Peer-reviewed literature databases (PubMed/Ovid Medline)	18 (34.0)
Grey literature (organizational websites, reports, meeting documents, strategic documents)	35 (66.0)

#### 2.4.2 Qualitative component

An interview guide was developed specifically for this study with open-ended questions (see online Supplementary file 2). Semi-structured interviews were conducted with 16 key informants, who are current/were prior members of EMR/Arab World public health-related networks during the data collection period (August 2024 – February 2026). All key informants received a consent form before participation, and interviews were scheduled according to the participants’ availability. After obtaining verbal consent, the interviews were conducted using preferred online platforms (Teams or Zoom) (26, 27). Ten interviews were conducted in English, while six were conducted in both Arabic and English. All interviews were conducted by the second author, who is an experienced public health professional with good knowledge of public health challenges and networks in the region. Interviews lasted between 30 and 120 min and were audio recorded after securing consent. Each interview was transcribed verbatim within one week by one member of the research team. The accuracy of the transcriptions was reviewed and corrected, as necessary, by the other members.

### 2.5 Data analysis

#### 2.5.1 Desk-based review

We descriptively analyzed the 53 retrieved documents by predefined themes to examine evidence: network objectives and main themes, network milestones, membership, geographic scope, and timeline. We used these data to enhance the interpretation of the qualitative findings.

#### 2.5.2 Qualitative component

Thematic analysis was conducted on the data collected through the KIIs. The research team first transcribed all sixteen interviews verbatim in the language of interview and subsequently translated transcripts into English for analysis. To ensure anonymity, each interview was assigned a unique number from 1 to 16. Inductive thematic analysis was then used to identify patterns within the qualitative data (28), supported by Dedoose software (29, 30) and following the Braun & Clarke framework (31) for thematic analysis. This framework includes six steps: (1) familiarization with data; (2) generation of initial codes; (3) reviewing themes; (4) defining themes; (5) naming themes; and (6) producing the final report. Responses were coded, breaking them down into similar concepts and ideas. The team subsequently cross-checked the coding and discussed discrepancies. Any disagreements were discussed and resolved by consensus. Axial coding followed, organizing emerging concepts into themes and categories. The themes and categories were documented in a Microsoft Excel spreadsheet (32). Finally, quotes used in this manuscript were returned to participants for validation, to ensure accuracy and avoid any loss of contextual meaning during interpretation.

### 2.6 Ethical considerations

Research ethics approval was obtained from the Institutional Review Board (IRB) at the American University of Beirut (AUB) during August 2024 (reference # SBS-2024-0261). The consent form, included in the invitation email to participants, outlined the study’s purposes, procedures, voluntary nature, right to withdraw, anonymity, confidentiality, and request to record interviews. Before each interview, the IRB-approved consent script was narrated, the participant was ensured to understand it, and verbal consent was obtained. The interview tool did not collect any personal identifying information. Participant confidentiality was maintained by using numerical codes in all diary notes, transcripts, analyses, and quotations. Data was kept confidential and accessible only to the research team for study purposes.

## 3 Results

### 3.1 Desk-based review results

As mentioned above, the included networks in our study could be categorized into three types, based on their purpose and structure. These are: networks of individuals, networks of institutions, and both.

#### 3.1.1 Individual-based public health-related networks

These networks are associations of individuals/professionals, such as specialists sharing common interests. They are dedicated to promoting public health by leveraging the expertise of their members. They provide a space to produce research, inform policy, exchange information, and advocate for education and research agendas. One of the pioneering networks is the Reproductive Health Working Group (RHWG), founded during 1988, to support multidisciplinary research network for Arab countries and Turkey. Described by its members as a capacity-building network, RHWG brings together experts from anthropology, economics, medicine, midwifery, nursing, sociology, population science, public health, and other fields, most of whom are women (33–38). RHWG’s mission is threefold: to generate contextual knowledge about women’s reproductive health in the Arab world and Turkey; to strengthen capacity for rigorous research; and to inform policies and practices in the region (34). While research has documented the network’s successes more than its challenges, Giacaman et al. highlighted that the political situation in the region has been a main restricting factor for network members traveling to meet and network (33).

Another individual-based network is the Arab Forum for Social Sciences and Health (AFSSH), which was one of the first individual networks, active from 2002 to 2018. What made it exceptional during that era was that it included not only health/biomedical scientists but also social and behavioral scientists in the Arab world. Unfortunately, we have not found online publications about this Ford Foundation-funded forum, hosted by the University of Balamand in Lebanon, except for a mention of its contribution to the development of anti-smoking legislation (39).

The Arab Public Health Association (ArPHA) was established in 2014 to strengthen the public health profession by defining standards, strengthening practice, and supporting all public health professionals, organizations, and those from all disciplines who contribute to the population’s health in their practice, work, and endeavors. ArPHA organized several regional conferences and maintains a very active WhatsApp group for its members. It issued an official statement addressing the public health consequences of the earthquakes in Turkey and Syria, contributed to advocacy on the impact of war and displacement on the well-being of women and girls in Gaza, and organized regional knowledge-exchange initiatives, including the COVID-19 webinar “Exit Strategy: The ‘New’ Normal” (40).

The Eastern Mediterranean Association of Medical Editors (EMAME) was established as part of the World Health Organization-Eastern Mediterranean Regional Office (EMRO) (41, 42). This followed the first and second regional conferences on medical journals held in Cairo in 2003 and Riyadh in 2004, respectively (43). EMAME aims to advance e-healthcare by disseminating high-quality scientific medicine, improve editorial standards and professionalism in medical editing, encourage research in peer review and medical editing, and provide continuing education for medical journal editors through regular conferences and courses (43–48). EMAME conducted several conferences and capacity building activities during the past 20 years. EMAME cooperates with the World Association of Medical Editors (WAME), the Forum for African Medical Editors (FAME), and other international associations in the field of medical journal publishing.

The Middle East and North Africa (MENA) chapter of the International Epidemiological Association (IEA) models individual-based public health networks within a global system. IEA/MENA aligns with the IEA’s overall objectives, such as facilitating communication among researchers and educators conducting epidemiological studies, and promoting epidemiology across various health fields, including social, community, and preventive medicine. These goals are achieved by hosting scientific regional meetings, as well as contributing to the International Journal of Epidemiology (IJE) and books like the Dictionary of Epidemiology, among other publications. IEA/MENA also offers workshops on scientific writing and outbreak investigations, as well as collaborative sessions on disease prevention, conflict resolution, and health systems (49, 50).

#### 3.1.2 Institution-based public health-related networks

Public health networks can also comprise institutions, including educational and professional organizations, that collaborate to address challenges in health improvement. One example is the Eastern Mediterranean Region Academic Institutions Network (EMRAIN), a network of public health institutions in the region which is currently inactive. EMRAIN was formally launched in March 2010, supported by WHO-EMRO, following a recommendation made at a consultative meeting in 2009 (51). The EMRAIN secretariat was hosted by the Faculty of Health Sciences at AUB, and the network included 11 EMR/Arab World public health institutions and three collaborators. The increase in public health programs in the EMR region, along with critical gaps in some countries, highlighted the need for collaboration among public health institutions to address these challenges. Consequently, the primary purpose of EMRAIN was to amplify the collective voice of academic public health institutions, enabling them to work together and become respected players in public health and health systems policy reform at the regional level (51). During its active years, EMRAIN did not limit its work to public health education. Instead, it created a sustainable platform for dialogue among all partners concerned with public health and health system strengthening, especially policymakers (52).

More recently, another institution-based network, the Evidence and Data to Policy (NEDtP), was established in 2019 by the WHO/EMRO as part of a framework for “improving national institutional capacity for use of evidence in health policy making in the Eastern Mediterranean Region” (53). The network currently includes 30 institutions, representing all 22 EMR countries. The primary objectives of the network are to support evidence-informed national health policy-making and to enhance regional and national technical capacity for utilizing evidence in health policy development (54–62). Additionally, one of the most significant achievements is developing a guide on evidence, policy, and impact for evidence-informed decision-making, which helps foster closer collaboration across different workstreams of the evidence ecosystem (60–62).

The Eastern Mediterranean Public Health Network (EMPHNET), a public health nonprofit organization, was established in 2009 to endorse better health in the EMR and beyond, and is considered a network of Field Epidemiology Training Programs (FETPs) in the region. EMPHNET’s goals include preventing and controlling diseases by strengthening the public health workforce, conducting operational research, enhancing public health programs, and promoting knowledge exchange and networking. It works with ministries of health, public health agencies, academic and research institutions, the private sector, international organizations, including United Nations agencies, community organizations, among others (63). EMPHNET is unique in its vast documentation of successes. Among the most significant is its ongoing effort to strengthen the public health workforce in the EMR, which it achieves by supporting and coordinating the work of FETPs in the region (63–74). EMPHNET has, to date, supported over 15 countries and implemented more than 35 FETP tiers.

#### 3.1.3 Individual and institution-based public health-related networks

The Arab Council for the Social Sciences (ACSS) is the only network, in our study, based on both individual and institutional membership, with the aim of strengthening the social sciences in the Arab region. ACSS’s objectives are to identify and address the needs of social scientists and their communities, enhance the capacity of researchers and institutions, promote independent and high-quality research, and provide forums for exchange and communication (75–79). The ACSS has organized seven thematic and methodological working groups, including groups focused on violence in the Arab region, critical security studies, ethnography, regionalisms, research on the arts, gendering the archive, and safeguarding knowledge production. In addition to fellowships and working groups, ACSS has actively advanced initiatives such as the Arab Public Data Initiative, the Critical Humanities Initiative, the InterAsia Partnership, Mapping the Production of Knowledge on Women and Gender, and New Research Agendas on Gender, thereby strengthening cross-program collaboration and impact (80–84).

### 3.2 Qualitative interviews results

A total of 16 key informants participated, comprising eight females (50%) and eight males (50%). Six participants reported holding doctoral degrees only, seven holding both an MD and a doctoral degree, two holding an MD and a master’s degree, while one reported holding a master’s degree only. Fourteen out of the 16 informants had over 25 years of experience, while two had approximately 15 years. Additionally, fourteen participants (87%) reported involvement in one or more of the defined regional public health–related networks, while two participants (13%) preferred to discuss networks in general without referring to a specific defined network, citing even networks that were not included in this research. Several participants belonged to multiple networks. Through analysis, seven themes and 31 categories were identified (see online Supplementary file 3). Furthermore, Supplementary file 4 presents data from the desk-based review that supported the qualitative findings.

Through analysis, seven themes and 31 categories were identified (see online Supplementary file 3).

#### 3.2.1 Theme 1: why networks exist and what they are expected to achieve?

Thematic analysis identified five main categories that define the aims and objectives of networks and why they exist: (1) networking; (2) capacity building; (3) research and publications; (4) policy and advocacy; and (5) health services.

Key informants most often cited networking as the primary reason for networks’ existence. It is essential for building connections among individuals and institutions in the region, facilitating interactions at annual meetings, conferences, and forums, sharing achievements, discussing public health priorities, and co-designing strategies relevant to the region. Of these networks, AFSSH was recognized as a key site for interdisciplinary dialogue, especially between social and behavioral scientists and biomedical scientists. One informant reflected on AFSSH’s role:

*“I think it was a priority for that association or forum to connect people to each other. So, one: they wanted to connect people from public health, medical, and nursing backgrounds with people from the social sciences and others. So, I think that was a very clear objective…”* – KII 13.

Another recurring category was capacity building to address workforce gaps, institutional instability, and uneven technical capacity across countries. Eight participants (50%) across six networks emphasized this. For instance, EMAME was described as a platform for training medical journal editors and advancing best practices in scientific publishing. On the other hand, NEDtP focused on building technical capacity for evidence-based, informed policymaking. It used targeted training to strengthen institutional support for the development of national health policy. Meanwhile, one key informant recognized EMPHNET’s role in partnering with ministries and institutions to address workforce gaps and systems-level challenges.

*“EMPHNET is a regional network in the Eastern Mediterranean Region… and it works to promote public health and to support countries in the region in strengthening their public health capacity—working mainly on boosting public health services and also strengthening the public health workforce. So, EMPHNET has attracted, over time, more attention and more focus from the different partners in the region.”* – KII 5.

Key informants perceived research and publications not only as a means of generating knowledge but also as a way to gain visibility, authority, and a regional voice. Six participants (37%) referenced six distinct networks: EMAME, focusing on medical editors; RHWG, supporting early-career scholars in all stages of reproductive research; AFSSH, blending public health research with social and behavioral aspects; the MENA Health Policy Forum, focusing on health policy exchange; ACSS, funding research; and IEA/EMR, providing a platform for presenting epidemiological research and supporting junior researchers at regional and global meetings.

*“Then the third function is that we (ACSS) are like a funding body because we give out grants and fellowships for individuals and for institutions to conduct research… So that’s why I’m calling it a hybrid institution”* - KII 15.

Moreover, policy and advocacy were examined in depth by three participants (19%). However, networks pursued different advocacy models depending on political access, mandate, and issue area. One participant mentioned the Palliative Care Network from outside the list of identified networks as an example of a network that stands out for its linkage of clinical practice to legislative advocacy, focusing decisively on critical issues such as end-of-life care and access to certain painkillers (including morphine). AFSSH, conversely, grounds its advocacy in community needs and strategically utilizes academic research to inform health policies, such as anti-tobacco legislation. One key informant directly described how NEDtP advocates embedding systematic, routine evidence production within ministries of health, ensuring evidence informs policy formulation:

*“It is essential to institutionalize structures and processes within the health system, particularly within the Ministry of Health, so that whenever policymakers need to make decisions, they are required to consider the available evidence as part of the policymaking process.”*– KII 14.

Lastly, the theme of health services was represented in the accounts of only three informants (19%). They referred to AFSSH and ArPHA. This limited emphasis on service delivery suggests that networks operate primarily at the coordination, policy, and knowledge levels, focusing less on direct implementation and on responding to community-level health needs.

#### 3.2.2 Theme 2: how partnerships shape the functioning of regional public health-related networks?

Thematic analysis identified five central categories that characterize the nature and diversity of partnerships of networks. These categories include partnerships with: (1) the public sector; (2) international organizations; (3) academia; (4) civil society; and (5) peer associations. Across networks, partnerships were portrayed not merely as collaborative arrangements, but as critical mechanisms shaping legitimacy, access to resources, and the ability to influence policy and practice.

Most participants (69%) indicated that partnership with the public sector is seen as strategically vital for advancing public health agendas, shaping policy, and ensuring active governmental participation in regional initiatives. Networks such as EMPHNET and NEDtP have formal and institutionalized ties with ministries, particularly the ministries of health. Others, like AFSSH and EMAME, reported more occasional engagement. These more fluid relationships were often shaped by factors such as political turnover, shifting leadership priorities, and the absence of formalized mechanisms for collaboration. Participants suggested that the degree of institutionalization of public sector partnerships directly shaped networks’ policy influence and continuity.

*“At every conference we hold… We always look at public-private relations. There was always a presence from specific ministries… to understand the research, to develop policies… even if it was related to the economy. We brought someone from the Ministry of Finance, because ultimately, someone has to fund health, economic, and social improvements… So, we always sought public-private relations… especially during conferences, so that they could be present and see what was happening.” – KII 9.*

Eight participants (50%) emphasized that partnership with international organizations such as the WHO, the United Nations Children’s Fund (UNICEF), and the United Nations Relief and Works Agency for Palestine Refugees in the Near East (UNRWA) is foundational. These partnerships facilitate technical support, enhancing the network’s visibility both regionally and globally. WHO emerged as the most frequently cited international partner. It was often credited with initiating or sustaining collaborations, whether through formal affiliations or capacity-building roles. However, the nature of these partnerships varied significantly. Some collaborations were structured and goal-aligned, characterized by ongoing joint projects and shared objectives. In contrast, others were more transactional or symbolic, limited to observer status or periodic funding support without deeper engagement or shared planning.

On the other hand, eight participants (50%) reported a range of academic engagement across networks, distinguishing formal from informal approaches. For example, EMRAIN is explicitly composed of academic institutions, with universities serving as foundational members and primary partners. In contrast, networks such as EMAME and RHWG engage with universities for specific purposes, including joint scientific events, editorial collaboration, and capacity-building workshops. ACSS further demonstrates formal partnerships through Memoranda of Understanding (MoUs) with over 20 universities, enabling structured, long-term collaboration in training, research, and convening activities. Conversely, some networks illustrate a more organic partnership model, rooted in enduring intellectual relationships between scholars rather than formal agreements.

Additionally, five participants (31%) valued civil society partnerships for implementing programs, reaching communities, and grounding public health interventions in local realities. Six participants (37%) discussed partnerships with peer associations operating at a similar organizational level, either regionally or globally. EMPHNET, for instance, is embedded within a global system of regional public health networks, named Training Programs in Epidemiology and Public Health Interventions Network (TEPHINET), and aligns with counterparts such as APHNET in Africa and similar initiatives in Southeast Asia and the Americas, supporting both regional collaboration and integration into global health dialogues. In contrast, ACSS exemplifies cross-regional collaboration by partnering with peer social science councils, such as CODESRIA in Senegal and CLACSO in Latin America, to strengthen South–South knowledge exchange.

#### 3.2.3 Theme 3: what has worked well in regional public health-related networks?

Thematic analysis identified several categories reflecting network success: (1) institutionalized capacity building; (2) coordinated public health crisis responses; (3) regional research and knowledge generation; (4) health service delivery contributions; (5) evidence-informed policy impact; and (6) national institutionalization. Key informants agreed that network success was measured by sustained initiatives rather than one-off or short-term activities.

Participants emphasized capacity building as the main success story of regional networks, with EMPHNET recognized for its institutionalized efforts, seen as a key marker of success. Embedding training within national and regional systems enabled these initiatives to endure beyond individual projects, expand across countries, and be replicated over time. Specifically, EMPHNET’s International Academy of Public Health (IAPH) and its regional Field Epidemiology Training Programs (FETPs) have provided hands-on, practice-based training for thousands of public health professionals. As one key informant described:

*“I think one prominent achievement is the Field Epidemiology Training, which is known as FETP, and this is why it has spread now. This is a different duration… So, training includes short-, medium-, and long-term courses, up to 2 years of field epidemiology training, and the beauty of this program is that it’s practice-based — it’s not just classroom. It supported different countries in preparing an epidemiology workforce to tackle* var*ious epidemics and health problems, and the program is gaining momentum.”* – KII 5.

Responding to public health crises was discussed by seven participants (44%). They noted that networks were effective during crises because pre-existing coordination enabled rapid mobilization, positioning them as standing platforms rather than *ad hoc* responders. For instance, the IEA’s link to the Global Outbreak Alert and Response Network (GOARN) enables rapid expert deployment whenever outbreaks emerge, speeding up the mobilization of epidemiologists and technical experts. EMPHNET’s operational work in conflict-affected regions, including Gaza, Sudan, Syria, Somalia, and Yemen, was also mentioned, where it scaled up interventions to strengthen public health systems under pressure. ArPHA also responded to the Gaza war, as described by one key informant:

*“The war on Gaza, we wrote to the UN Secretary General. We have written to some heads of state, in Britain, for example, and some of the Arab countries, we wrote to heads of state. On issues of Gaza, we asked international organizations to support Gaza humanitarian efforts”* – KII 16.

Seven participants (44%) described research as essential for building the networks’ scholarly knowledge and advancing regional evidence. Achievements included exposing neglected regional issues, integrating local experience into global public health debates, and documenting the social and political determinants of health in fragile contexts, particularly in Palestine. Examples include the RHWG’s research paper on “Health Research in a Turbulent Region: The Reproductive Health Working Group” (43), the collaborative work of EMPHNET with governments to address the COVID-19 epidemic (66), and the plan of ArPHA to issue an Arab Journal of Public Health.

Policy change was discussed by three participants (19%). Although mentioned less frequently, policy change was viewed as a significant indicator of success, reflecting the depth, credibility, and maturity of networks capable of influencing national decision-making processes, illustrating how certain networks extended their work beyond research and knowledge sharing to influence national policies, tackling critical public health issues such as anti-tobacco consumption legislations and maternal and child health, supported by research evidence and regional dialogue. National institutionalization was mentioned in the establishment of national entities, which was discussed by two participants who reflected on IEA/MENA’s role in supporting the creation and engagement of national public health and epidemiological associations. This was particularly successful in countries such as Egypt, Iran, Lebanon, Saudi Arabia, and Tunisia, where national societies were either established, fostered, or actively engaged.

#### 3.2.4 Theme 4: why do regional public health-related networks struggle or fail?

Thematic analysis found seven key causes of network failure: (1) funding; (2) governance and leadership; (3) political and social context; (4) human resources; (5) lack of institutional continuity; (6) conflicting individual and institutional interests; and (7) linguistic fragmentation. Participants stated that network failure results from combined financial, organizational, political, and human resource constraints, not a single cause.

Almost all participants (94%) agreed that the lack of sustained, flexible financing is the most destabilizing challenge. They emphasized that most regional public health networks are largely dependent on donor funding: when external support ends, or donor priorities shift, “things more or less fall apart- KII 1.” The absence of institutionalized fundraising mechanisms, such as stable in-country and regional sources or internal revenue strategies, further exacerbated this instability. Participants clarified that funding challenges extend beyond technical aspects, such as insufficient funds or unreliable funding streams, and involve political dimensions in which donor-driven agendas may conflict with local or regional priorities. Additionally, they reported that funders often struggle to understand or value network activities, given the perceived high costs and indirect impact of maintaining such networks. Participants framed funding not merely as a technical constraint but as a structural vulnerability that shapes networks’ priorities, continuity, and long-term viability.

*“Networks are very hard to raise funding for because, for funders, they do not see the direct impact of it. For network members, it’s nice because they come together, meet people, and advance their ideas… But it’s hard to say what the concrete outcome of a network is. And it usually—networks—requires a lot of travel funds, so they are expensive because people have to get together. One thing—you have to get together—and that’s expensive… So, I know that over my lifetime, I’ve tried to raise money for networks many times, and it’s difficult. It’s much easier to raise funding for a specific research project or a specific activity that has a certain impact.”* – KII 7.

Eight participants (50%) identified governance and leadership as key challenges, highlighting tensions within network structure and leadership. Many networks depended on individual leaders rather than institutions, leaving them vulnerable to role changes, burnout, or shifting priorities. Some participants stressed that formal governance, mandates, bylaws, and clear roles are vital for continuity, legitimacy, and organizational memory. Others warned that rigid structures could stifle creativity, create conflict, and add bureaucracy. This tension shows the recurring dilemma in network design: balancing the flexibility and energy of individual leadership with the need for institutional continuity and accountability. One participant reflected on sustaining a network without a dedicated leader:

*“Really, to run a network well, you need a full-time coordinator… and I was doing it on top of everything else… I was (an administrator) … I was doing research… I was teaching, and I was doing this on the side… So you know, it’s really tough to find the time, and I did it because I valued it. And that’s again related to the funding because if you do not have funding for a full-time coordinator, then the person has to squeeze it into their time, and the steering committee, it was the same thing; they were doing this on top of everything else that they do.”* – KII 7.

Nine informants (56%) cited political and social challenges, including fragmentation, inequities, and instability that limit regional public health collaboration. AFSSH, the most prominent example, faced political and logistical hurdles leading to its dissolution. Participants noted geopolitical tensions, visa restrictions, governance gaps, and social conflicts as major barriers. Fragmentation in the Arab world also hindered pan-Arab associations, worsened by competition for leadership, representation, and language.

*“When we talk about an Arab association, again, it’s not straightforward because if you look at the Arab world now, it’s totally broken, broken down into… it’s shattered tactically… So you talk about an Arab association… what does it mean? You put Syria, Iraq, Saudi Arabia… and Sudan and Libya… Gosh… so again we have our problems… We do have our problems, so we know that there are challenges…” – KII 13.*

Seven key informants (44%) discussed challenges in human resources, specifically highlighting a reliance on a small group of highly committed individuals, a lack of institutional commitment or formal staffing, sporadic participation tied to funding availability, and diminished engagement among younger professionals who are either emigrating or less interested in regional efforts. These findings point to weak institutional anchoring and over-reliance on voluntary engagement, rather than a lack of commitment. One participant described:

*“A second thing is, of course, the non-sustainability of these members… People come and go, and because of the non- formality, you do not have… something that ties them to the network…. There’s nothing binding for members, so as they are available, they support; as they become busy or move on to different places, they are no longer there… So, sustainability is always an issue. This comes with a lack of accountability also…”* – KII 2.

Moreover, six key informants (37%) emphasized that the lack of institutional continuity undermined network sustainability. Key findings included networks experiencing fluctuating momentum with bursts of activity and periods of complete dormancy. Informants attributed this instability to factors such as political instability, leadership turnover, a lack of structured follow-up, weak institutional anchoring, and emergencies like the COVID-19 pandemic. Four informants (25%) highlighted conflicting individual and institutional interests as a key barrier. These included tensions over network leadership, perceived dominance by certain institutions, and competing personal agendas. Specifically, some networks were seen as monopolized by a few countries or institutions. Informants described instances where individuals prioritized advancing their own or their institution’s interests, creating competition for resources that undermined overall collaboration. As one participant recalled:

*“But what ended it was… it was a network of individuals, not institutions… OK. So that’s, I mean, I think one has to kind of think about what kind of network one wants, whether it’s a network of individuals or a network of institutions. And I mean, I think what ended up happening is that some of the individuals involved saw this as a way to build up their positions in their institutions… So they saw it as a way of fundraising for their institutions, and that, I think, was the main thing that undermined it—they had their own agendas, basically as individuals, and they were thinking mostly of their own power within their institutions, and that was not what the network was designed to do.”* – KII7.

Three key informants (19%) cited linguistic fragmentation as a significant challenge. Linguistic diversity, beyond widely spoken Arabic and English, such as French, Persian, and Urdu created logistical and conceptual barriers to effective network operation. Language barriers compounded existing inequalities in participation and influence within networks.

#### 3.2.5 Theme 5: what is needed to sustain regional public health-related networks?

Thematic analysis identified eight categories reflecting participants’ recommendations for sustaining networks, including (1) governance and leadership arrangements; (2) mechanisms for sustained communication and coordination; (3) sustainable and diversified financing; (4) strategic planning mechanisms; (5) human resource sustainability; (6) incentives for sustained engagement; (7) understanding the political and social context; and (8) building trust.

Building on these recommendations, nine participants (56%) emphasized the need for institutional, rather than individual, leadership to ensure network sustainability. They advocated establishing secretariats, developing clear mandates, and rotating leadership roles to distribute responsibility and preserve continuity, while cautioning that over-formalization could create unnecessary bureaucracy. Two participants (13%) noted that formal structures should only be adopted as needed and after clarifying the network’s goals.

*“And then being able to establish a technical secretariat or technical body because, in my opinion, there is no network or anything that can succeed without people who are dedicated. Even if it is a small number of people, if they are dedicated to working in this area, it will succeed. But if people are all part-time and they do not have time and they just come for meetings, then they will meet, they will discuss, and then they will get apart and then forget about things until they come back…”* – KII 5.

Key informants (69%) recommended combining digital and face-to-face networking to improve collaboration and sustainability. Participants emphasized that sustained communication mechanisms are essential for maintaining engagement, institutional memory, and collective momentum. Digital platforms like Zoom, WhatsApp, email, and social media were seen as cost-effective, especially after the COVID-19 pandemic. Face-to-face meetings remained important for building relationships and trust. Newsletters, documentation, membership databases, and regional observatories were also suggested to enhance visibility, track progress, and foster institutional connections.

*“Sustainability requires regular programs and meetings to keep stakeholders informed and engaged. Providing updates, feedback, and continuous communication helps maintain momentum. Regular interactions also strengthen links with Ministries of Health and across national entities, bringing them closer together and enhancing collaboration”* – KII 14.

Sustainable financing was prioritized by ten key informants (62%), who advocated for robust, consistent financial models. Five participants (31%) proposed membership fees, drawn from individuals or institutions, to promote ownership, accountability, and operational stability while limiting reliance on external donors. Three participants (19%) referenced international models from the U.S. and Europe, where fee-based approaches are used to secure financial independence and facilitate long-term planning. Four participants (25%) also saw financing models as a way to reduce dependence on external donors and strengthen ownership among members.

Eight participants (50%) stressed the importance of strategic planning by articulating clear objectives, structures, and evaluation criteria. Two participants (12%) noted that networks flourish when incorporated into formal strategies with defined accountability. Another two underscored the need for measurable targets and for adopting frameworks such as the Theory of Change to prioritize results. Several participants observed that broad consultations and participatory planning before launch can clarify structure, align with community priorities, and guide adaptive design. When planning, seven key informants (44%) emphasized the importance of defining the network’s scope. They focused on positioning, responsibilities, and priorities. They cautioned that an overly broad mandate could dilute focus and urged a defined strategic path.

*And because we were very broad. You know, across all the social sciences, across all the regions, it was quite complicated… If an association or an organization is focused more narrowly on a theme or on a discipline or on a particular field and so on… it will be a little easier…but we are very committed to interdisciplinarity and to addressing a wide range of issues”* - KII 15.

The critical role of human resources in sustaining public health networks was emphasized by six key informants (33%). Networks often thrive on the passion and voluntary engagement of a small group of highly committed individuals. However, this individual-driven model is fragile. Momentum can be lost when key actors retire, shift focus, or lose institutional backing. To address this, participants stressed the importance of intentional leadership renewal through capacity-building initiatives. These initiatives prepare future network leaders and strengthen collaborative skills. It was also recommended to promote intergenerational exchange and engage young professionals. Such strategies help maintain coherence while enabling renewal and broader participation. In addition, one participant highlighted the importance of including policymakers and policy developers alongside researchers.

The importance of incentives in motivating continued participation in networks was emphasized by five participants (31%). Incentives were framed as mechanisms to sustain engagement, rather than as individual rewards. Financial, professional, and intellectual incentives were seen as essential. These included awards, scholarships, training, increased visibility, collaborative research, accreditation benefits, and access to knowledge and tools. A few noted that symbolic recognitions can help contributors feel valued. Another participant emphasized the importance of establishing a collective sense of purpose early. This should occur before members begin to question their personal benefits.

Finally, two participants (12%) emphasized that building trust, through transparency, openness, and consistent communication, is especially important in regions where mistrust is common. This trust must be actively reinforced. Trust was described as both an outcome of sustained engagement and a prerequisite for effective collaboration. One participant also noted that understanding the political and social context is crucial, as networks must remain aware of their operating environment.

## 4 Discussion

This qualitative study provides in-depth insights into the experiences of public health-related networks in the EMR and Arab world. It examines factors that facilitate or hinder their functioning and identifies best practices to inform the design of sustainable and resilient networks. The study draws findings from sixteen key informants who are or were members of regional networks. It contextualizes these insights through a desk-based review, noting that the review only yielded reports that show the activities of these networks and did not offer an analysis of what has worked or not, emphasizing a critical gap in the literature. The literature contains multiple definitions of a “Network” (6, 85). Nevertheless, this paper does not address the lack of consensus on terminology, which complicates the use of networking approaches. Instead, the methodology builds on the premise that a public health network does not require to be formally titled as a “network.” Rather, it refers to organizational forms comprising systems of interconnected actors that aim to achieve common public health goals (4).

The findings revealed that actors establish such networks when they recognize gaps in regional collaboration and desire interaction. Diverse governance models, membership tiers, partnership patterns, and socio-political realities shape their trajectories. Interviewees’ historical accounts echo the literature’s portrayal of networks as responses to interdisciplinary isolation. For example, leaders formed EMPHNET to consolidate scattered expertise among national FETPs and respond to public health challenges in the region (66, 67, 86). International associations share similar experiences. Founders established the International Association of National Public Health Institutes (IANPHI) in response to SARS, avian influenza, and Ebola (87, 88). The qualitative findings also highlighted the emotional investment of the founding members. This supports the idea that individual agency, rather than institutional mandates, often sparks initial momentum for such networks. One example is RHWG, which a group of scholars and practitioners founded after meeting and working informally for several years and gradually building a formal structure (35, 36).

Literature categorizes public health-related networks into health service delivery (8), such as EMPHNET, which works on the ground as a trusted partner (70), policy networks, such as NEDtP, which serves as mechanisms for evidence-informed health-related policies and system change (89), or research networks, such as such as IEA/MENA, which produce studies of varying scales (4). Nonetheless, our study found that capacity building and workforce development can be standalone themes. They contribute not only to the categories above but also independently. ACSS demonstrated this by supporting 413 grantees and fellows through 31 programs since 2008 (75). On the other hand, policy influence appeared as a central theme in the literature (42, 90, 91), not necessarily emphasized as such by key informants. This suggests that policy engagement, while important, may occupy a less prominent position in the operational priorities of the assessed regional networks. Divergence exists between stated objectives and on-the-ground practice.

Our study’s results highlight the centrality of partnerships with the public sector, international organizations, and academia. These results mirror patterns reported in other regional contexts. For example, the European Public Health Association (EUPHA) maintains deep governmental linkages (92), and the South-East Asia Public Health Education Institutions Network (SEAPHEIN) actively collaborates with WHO (93). Literature similarly identifies partnerships with civil society as important but less formalized. For instance, it suggests that a bilateral relationship between networks of schools of public health and civil society organizations can be beneficial in building community partnerships (94). However, these partnerships often emerge from shared projects rather than long-term agreements (35, 91). Our findings show that political volatility and leadership turnover in the EMR frequently disrupt even well-established partnerships. This pattern reinforces the importance of flexible and multi-sectoral strategies. Additionally, our qualitative findings and desk-based review both indicate that actors in the region engage minimally with the private sector and think tanks, which suggests an untapped opportunity for diversifying influence and resources.

Our findings indicate that a significant amount of work today is conducted in public health-related networks. These networks are crucial to addressing the region’s grand challenges, as demonstrated by the success stories identified in both the desk-based review and qualitative findings. However, several key factors can cause networks to fail in their ambitions. First, a lack of sustainable resources, whether financial or human resources (4, 95), as noted in the qualitative findings and the literature review. Second, the absence of institutionalized fundraising mechanisms and reliance on external funding contribute to failures. Third, weak governance is identified as another risk. While literature does not prescribe specific leadership or governance tools, it highlights that the prominence and power of certain actors can become distorted, allowing members to steer the network’s direction for personal advantage (4, 96). Fourth, our study identifies dependence on leaders’ behavior as another failure factor (97, 98). Finally, the qualitative results reveal mixed views about whether formal governance, with clear mandates and bylaws, or informal governance should structure a network. The networks that endured, such as EMPHNET and ACSS, did so by institutionalising what fragile networks have left informal, such as diversified funding, structured governance, and leadership succession.

While reasons for failure seem to be numerous, our study revealed motives for sustaining public health-related networks. Indeed, network sustainability can also be a key focus, as significant investment is required for its maintenance and upkeep. Furthermore, recommendations for sustaining networks, as articulated by key informants, resonate strongly with the literature, particularly in calls for establishing clear goals and purpose early in network development (99), as well as maintaining a flexible governance structure (99) and ensuring skilled leadership by recognizing the unique role of public health professionals by governments and health systems (100, 101). However, the tension between flexibility and stability emerged as a recurrent theme and a key consideration for future network design. In addition, our study reveals a gap in the presence of public health networks that linked academic and research institutions, as exemplified by EMRAIN, which did not persist long enough to demonstrate what worked; this is notable, given that the need for such collaboration between public health academic/research institutions is strongly recommended (102, 103).

## 5 Strengths and limitations

To our knowledge, this maybe the first study to explore and compare the experiences of public health-related networks in the EMR and Arab world. Another strength of our paper is that it used a qualitative approach supported by desk-based review. Moreover, we included current active networks, as well as dormant and demised ones, and interviewed senior key informants from different entities and institutions, across the region.

There are three limitations to note. First, despite efforts to identify more networks through database searches and expert consultations, some regional networks may have not been identified. Second, although the qualitative component included participants from the various networks, informants for some networks were overrepresented in the sample, such as AFSSH which was represented by four informants. This may have resulted in those perspectives exerting greater influence on the findings. In addition, it was not possible to interview any key informants from the MENA Health Policy Forum. This imbalance in representation, may have influenced the findings regarding the functioning and operational challenges of regional health research networks. However, the semi-structured interviews achieved data saturation, enhancing the generalizability of the results. Additionally, qualitative research could potentially constrain ability to analyze differences in information from key informants with varied experiences.

Future research should pursue a mixed-method approach, including quantitative analysis based on the themes of this study to develop a tool that measures networks beyond their establishment phase, and qualitative research to collect feedback from network beneficiaries and identify clear indicators of success and failure. Lastly, future research could explore gender-based differences in network leadership and examine how these differences influence network priorities, thematic focus, and modes of operation.

## 6 Recommendations

Our study has several implications for sustaining public health-related networks in the EMR, the Arab world, and beyond. We present our conclusions and recommendations under six main themes.

### 6.1 Establishing a flexible governance model

Our study showed that sustainable secretariats, clear mandates, and rotating leadership roles can reduce reliance on individual authority and agendas. These steps help prevent dominance by any member or organization. However, it is recommended to strike a balance between formalization and avoiding excessive rules that may hinder innovation and create bureaucracy.

### 6.2 Ensuring sustainable financing

The path to sustainable financing remains uncertain. Our study found that fundraising for networks is challenging, highlighting the need to fund through targeted programs. Second, securing membership fees is a useful method, similar to models in other regions, as it improves commitment of paying members, holding them more accountable to the network. Investing in philanthropic activities using domestic resources, with a strategic rather than *ad hoc* approach, is recommended. This could help diversify funding sources and render networks less reliant on a single resource, thereby increasing their resilience.

### 6.3 Setting tangible goals, but also monitoring them

Strategic goals and objectives are often set during a network’s development phase. However, they are less often revisited in the operational phase. Our study showed the importance of having clear purpose and evaluation mechanisms with clear accountability lines. It is also vital to reassess the network’s purpose and objectives periodically. Doing so helps networks respond effectively to the dynamic challenges within the public health sector.

### 6.4 Sustaining long-term momentum

One key informant said, *“Because if the network is not reminding you of itself, it’s almost like it does not exist*.” Networking requires ongoing efforts and the use of various communication tools, both virtual and in-person, to keep momentum. Ensuring continued communications via creating databases, newsletters, and observatories, as well as using social networking platforms such as WhatsApp and LinkedIn, is essential to maintain network memory. Network effectiveness improves with a well-coordinated process acknowledged by all members and partners.

At the same time, nowadays the most effective channels for linking professionals (including public health scholars) are often social networking platforms, e.g., WhatsApp groups, LinkedIn, and ResearchGate, which enable continuous visibility and interaction.

### 6.5 Investing in the younger generation

Our study highlighted the importance of investing in human resources as essential for network sustainability. It also emphasized the value of new generations and fresh perspectives for renewal and intergenerational exchange. The “strength of weak ties” theory, prominent in network literature, proposes that important connections are often not the strongest. Instead, weaker ties connect previously unlinked groups through bridges (104). Even if new generations have weaker ties or less experience, they can serve as vital bridges, pumping new energy and fresh ideas within networks as they learn from senior members and carry the legacy of the network forward.

### 6.6 Demonstrating value and building trust

Our study focused on identifying issues and opportunities that add value for network members, their organizations, the network, and the broader public health system. Building trust requires transparency, openness, and consistent communication. This is especially crucial in regions where mistrust is common and must be actively addressed. Networks must remain aware of their context.

## Data Availability

The original contributions presented in the study are included in the article/Supplementary material. Further inquiries can be directed to the corresponding author.
