# Supplementary material for: What makes public health-related networks work? Qualitative insights from the Eastern Mediterranean and Arab world
Source: Front Public Health. 2026 Aug 3;14:1880808. doi: 10.3389/fpubh.2026.1880808 (PMC13478113; doi:10.3389/fpubh.2026.1880808)
Supplement: Supplementary file 1 [file Table_1.docx]

**What Makes Public Health-related Networks Work? Qualitative Insights from the Eastern Mediterranean and Arab World**

**Ahmad Mandil^1^, Maya Hassan^2^, Iman Nuwayhid^2*^**

^1^Epidemiology Department, High Institute of Public Health, University of Alexandria, Alexandria, Egypt

^2^Faculty of Health Sciences, American University of Beirut, Beirut, Lebanon

Supplementary Material – Search Strategy

## 1.1 Databases:

1. Medline/PubMed
2. Academic Search Premiere (EBSCO)
3. Google Scholar
4. Grey Literature

## 1.2 Search Strategy for each database:

### 1. *Medline/PubMed*

| **Concept** | **MeSH Heading** | **Keywords** |
| --- | --- | --- |
| Public health networks | N/A | (Health adj3 (community or public or polic* or research* or data or surveillance or informat* or system*) or epidemiolog* or (disease* adj2 outbreak* or communicable or noncommunicable) and (network* or association* or Forum* or group* or coalition* or institution* or school* or university* or alli*)).mp. |
| Eastern Mediterranean Region | Eastern Mediterranean Region/ or Arab World/ or Middle East/ or Africa, Northern/ or Afghanistan/ or Algeria/ or Bahrain/ or Comoros/ or Djibouti/ or Egypt/ or Iraq/ or Iran/ or Jordan/ or Kuwait/ or Lebanon/ or Libya/ or Mauritania/ or Morocco/ or Oman/ or Palestine/ or Qatar/ or Saudi Arabia/ or Somalia/ or Sudan/ or Syria or United Arab Emirates/ or Yemen/ | ((Mediterranean or middle) adj (east or region) or (Arab adj (countr* or world)) or (Middle East and North* Africa) or MENA or EMR or Afghanistan or Algeria or Bahrain or Comoros or Djibouti or Egypt or Iraq or Iran or Jordan or Kuwait or Lebanon or Libya or Mauritania or Morocco or Oman or Palestine* or Gaza or (West adj1 bank) or Qatar or (Saudi adj2 arabia) or Somalia or Sudan or Syria or (united adj2 emirates) or Yemen).mp. |
|  |  | (AFSSH or ACSS or ArPHA or EMAME or EMPHNET or EMRAIN or IEA or NEDtP or RHWG).mp. |

### 2. *Academic Search Premiere (EBSCO)*

| **Concept** | **Subject Terms** | **Keywords** |
| --- | --- | --- |
| Public Health Networks/Institutions | N/A | TI ( (Health N2 (community or public or polic* or research* or data or surveillance or informat* or system*) or epidemiolog* or (disease* N1 outbreak* or communicable or noncommunicable) and (network* or association* or Forum* or group* or coalition* or institution* or school* or university* or alli*)) ) OR AB ( (Health N2 (community or public or polic* or research* or data or surveillance or informat* or system*) or epidemiolog* or (disease* N1 outbreak* or communicable or noncommunicable) and (network* or association* or Forum* or group* or coalition* or institution* or school* or university* or alli*)) )  TI ( (AFSSH or ACSS or ArPHA or EMAME or EMPHNET or EMRAIN or IEA or NEDtP or RHWG) ) OR AB ( (AFSSH or ACSS or EMAME or EMPHNET or EMRAIN or IEA or NEDtP or RHWG)) |
| Mediterranean Region | SU ((Mediterranean Region/ or Arab World/ or Middle East/ or Africa, Northern/ or Afghanistan/ or Algeria/ or Bahrain/ or Comoros/ or Djibouti/ or Egypt/ or Iraq/ or Iran/ or Jordan/ or Kuwait/ or Lebanon/ or Libya/ or Mauritania/ or Morocco/ or Oman/ or Palestine/ or Qatar/ or Saudi Arabia/ or Somalia/ or Sudan/ or Syria or United Arab Emirates/ or Yemen/) | AB ( ((Mediterranean or middle) adj (east or region) or (Arab adj (countr* or world)) or (Middle East and North* Africa) or MENA or EMR or Afghanistan or Algeria or Bahrain or Comoros or Djibouti or Egypt or Iraq or Iran or Jordan or Kuwait or Lebanon or Libya or Mauritania or Morocco or Oman or Palestine* or Gaza or (West adj1 bank) or Qatar or (Saudi adj2 arabia) or Somalia or Sudan or Syria or (united adj2 emirates) or Yemen). ) OR TI ( ((Mediterranean or middle) adj (east or region) or (Arab adj (countr* or world)) or (Middle East and North* Africa) or MENA or EMR or Afghanistan or Algeria or Bahrain or Comoros or Djibouti or Egypt or Iraq or Iran or Jordan or Kuwait or Lebanon or Libya or Mauritania or Morocco or Oman or Palestine* or Gaza or (West adj1 bank) or Qatar or (Saudi adj2 arabia) or Somalia or Sudan or Syria or (united adj2 emirates) or Yemen) |

### 3. Google Scholar Advanced Search

| With all of the words | Public health |
| --- | --- |
| With the exact phrase | Eastern Mediterranean Region |
| With at least one of the words | Association network forum |
| Return articles between | 1980- 2024 |
| Search line | Public health association OR network OR forum "Eastern Mediterranean Region" |

### 4. Grey Literature

- The WHO Eastern Mediterranean Regional Office (EMRO) for reports and publications
- “WHO Index Medicus for the Eastern Mediterranean Region” (IMEMR)
- Meetings, conferences and workshops
