# Supplementary material for: What makes public health-related networks work? Qualitative insights from the Eastern Mediterranean and Arab world
Source: Front Public Health. 2026 Aug 3;14:1880808. doi: 10.3389/fpubh.2026.1880808 (PMC13478113; doi:10.3389/fpubh.2026.1880808)
Supplement: Supplementary file 2 [file Table_2.docx]

**What Makes Public Health-related Networks Work? Qualitative Insights from the Eastern Mediterranean and Arab World**

**Ahmad Mandil^1^, Maya Hassan^2^, Iman Nuwayhid^2*^**

^1^Epidemiology Department, High Institute of Public Health, University of Alexandria, Alexandria, Egypt

^2^Faculty of Health Sciences, American University of Beirut, Beirut, Lebanon

**Supplementary Material - Semi-Structured Interview**

## 1.1 Definition & Examples:

**Q1:** Can you please describe your regional public health network?

- Please elaborate on the foundation year, mandate, objectives, coverage, membership of your association/network.
- What other information can you provide to reflect on your network?
- Please share weblink to your website (as applicable).

## 1.2 Partnerships & Collaborations:

**Q2:** What partnerships does/did your association/network have with different sectors? Please elaborate as much as possible.

- Public sector
- Private sector
- Non-governmental organizations
- UN agencies
- Individual professionals
- Other, specify

**Q3:** Have the mentioned association/network collaborated with other public health association(s)/network(s) within the EMR region or in other regions?

- If so, please provide detailed information.

## 1.3 Success stories:

**Q4:** What were/are some success stories of your association/network? Please elaborate as much as possible.

**Q5:** Please describe the role of your association/network in responding to public health crises in the region, as applicable?

- Humanitarian crises
- Natural disasters
- Infectious diseases outbreaks
- COVID-19 pandemic
- Other, specify

## 1.4 Challenges & Obstacles:

**Q6:** Can you discuss the challenges that have hindered the sustainability of your network or association?

- Human resources
- Financial resources
- Communication/networking
- Technical aspects
- Political readiness
- Other challenges, please specify

## 1.5 Recommendations:

**Q7:** Based on your experience, what recommendations and actions would you propose for sustainable public health networks/associations in the region?

- Governance arrangements
- Roles and responsibilities
- Financing
- Others, specify

**Q8:** Are there any lessons to learn from the experience of your public health association/network for other similar ones in the EMR region?

**Q9:** To the best of your ability, please provide examples of past or current public health association(s)/network(s) in the Eastern Mediterranean region (EMR).

**Q10:** Are there any additional concerns or issues you would like to talk about?

**Q11:** Do you have any questions for us?
