# Supplementary material for: What makes public health-related networks work? Qualitative insights from the Eastern Mediterranean and Arab world
Source: Front Public Health. 2026 Aug 3;14:1880808. doi: 10.3389/fpubh.2026.1880808 (PMC13478113; doi:10.3389/fpubh.2026.1880808)
Supplement: Supplementary file 3 [file Table_3.docx]

**What Makes Public Health-related Networks Work? Qualitative Insights from the Eastern Mediterranean and Arab World**

**Ahmad Mandil^1^, Maya Hassan^2^, Iman Nuwayhid^2*^**

^1^Epidemiology Department, High Institute of Public Health, University of Alexandria, Alexandria, Egypt

^2^Faculty of Health Sciences, American University of Beirut, Beirut, Lebanon

Supplementary Material - Summary of the qualitative analysis

| Themes | **Categories** | **Definition** | **Summary** | **Quotes** |
| --- | --- | --- | --- | --- |
| **Theme 1:** Why networks exist and what they are expected to achieve? | Networking | Captures how regional public health- related networks were created for connection, and collaboration. | Networking emerged as a central category, emphasized by eight key informants, as a tool for cultivating connections among individuals and institutions in the region. | *“I think it was a priority for that association or forum to connect people to each other. So, one: they wanted to connect people coming from public health, from medical background, nursing background, with people from social sciences and others. So, I think that was a very clear objective…”* – KII 13 |
|  | Capacity Building | Captures how networks were created to contribute to strengthening public health skills through trainings, workshops, collaborative meetings, and mentorship. | Eight participants highlighted such efforts across six key networks, EMAME, EMPHNET, NEDTP, AFSSH, RHWG, and ACSS, to strengthen professional skills, institutional capabilities, and collaborative learning across the region. | *"So, EMPHNET is a regional network in the Eastern Mediterranean Region… and it works to promote public health and to support countries in the region in strengthening their public health capacity—working mainly on boosting public health services and also strengthening the public health workforce. So, EMPHNET has attracted, over time, more attention and more focus from the different partners in the region."* – KII 5 |
|  | Health Services | Highlights how some networks contribute not only to research and policy, but also to improving or supporting public health service delivery. | Only two participants highlighted networks’ efforts in addressing clinical care gaps and community-level health needs in the region. | *"I'm also part of a network that supports the work around cervical cancer elimination. We call it the MENA HPV Forum, for example—which is also the MENA HPV Coalition—which brings together different partners that work to support cervical cancer elimination in this region…"* – KII 2 |
|  | Policy and Advocacy | Reflects how networks engage in influencing health policy, advocating for legislative changes, and contributing expert input to public health governance. | Three participants, referencing four networks: the MENA Health Policy Forum, the Palliative Care Network, NEDtP and AFSSH, declared that policy and advocacy is a main role of public health-related networks. | *“So, this means that you need to have structures, processes institutionalized within the health system… within the Ministry of Health… that, you know, for any question—when the policymaker wants to make a decision—he needs to consider… he is required, requested to look at… to consider… to consider the evidence for making the policy…”* – KII 14 |
|  | Research & Publications | Reflects how some regional networks were established to enhance research and publication efforts. | Six participants referencing six networks: EMAME, RHWG, AFSSH, the MENA Health Policy Forum, ACSS, and IEA, recognized the role of networks in advancing research in the region. | *"Then the third function is that we (ACSS) are like a funding body because we give out grants and fellowships for individuals and for institutions to conduct research... So that's why I'm calling it a hybrid institution"* - KII 15 |
| **Theme 2:** How partnerships shape the functioning of regional public health networks? | Partnership with the public sector | Examines cooperation with government agencies and public institutions responsible for health policy, regulation, and service delivery. | Most participants agreed that partnership with the public sector is the most dominant relationship. | *“At every conference we hold… We always look at public-private relations. There was always a presence from specific ministries… to understand the research, to develop policies… even if it was related to the economy. We brought someone from the Ministry of Finance, because ultimately, someone has to fund health, economic, and social improvements… So, we always sought public-private relations… especially during conferences, so that they could be present and see what was happening.”* – KII 9 |
|  | Partnership with international organizations | Includes cooperative relationships with global health bodies, such as the World Health Organization (WHO), UNICEF, or regional alliances. | Eight participants emphasized that partnership with international organizations, such as the WHO, UNICEF, and UNRWA, is foundational. | *“IEA is in official relations with the World Health Organization. WHO has a mechanism for working with various institutions globally, one of which includes 'official relations'—this enables exchange of experience, exchange of professionals, and collaboration during national, regional, and international crises. So, IEA is in official relations with WHO. This is a natural partner. We have also collaborated with UNICEF.”* – KII 1 |
|  | Partnership with academia | Captures joint efforts with universities, research centers, and educational institutions. | Eight participants revealed a spectrum of academic engagement across networks, ranging from structural integration to informal collaboration. | *“We are not formal partners with associations… But we have very strong relationships, particularly with the Faculty of Health Sciences at AUB. This is very important to us. Sometimes we think alike; sometimes we don’t—we argue… but we have forged intellectual and personal relationships. we grew up together and worked together on many things. Not all the time, but when we do, it’s based on shared interests. It’s not a formal partnership.”* – KII 10ss |
|  | Partnership with civil society | Refers to collaborations with non-governmental organizations (NGOs) to implement public health programs, deliver community-based services, and advocate for policy changes. | Partnerships with civil society were referenced by five participants. | *“They meant in complementarity. And then, it also needs downstream partnerships to those who are working on the ground—especially if you're a network that does not have much presence on the ground. You need to collaborate with civil society or communities who would be able to support your work implementation on the ground.” —* KII 2 |
|  | Partnership with peer organizations | Captures collaboration with other public health -related networks and associations. | Five participants talked about partnerships with peer associations operating at a similar organizational level, either regionally or globally. | *"Just a point maybe to add here, this is not only in the region, we also do cross regional collaboration…. so, we have a very good relationship with CODESRIA in Senegal, which is the Pan African Social Science Council…. We work with CLACSO based in Buenos Aires, which is the Latin American Social Science Council…. "* – KII 15 |
| **Theme 3:** What has worked well in regional public health networks? | Institutionalized capacity building | Refers to structured and informal efforts by networks to strengthen skills and mentorship. | Most participants emphasized capacity building as the main success story of regional networks. | *"I think one prominent achievement is Field Epidemiology Training, which is known as FETP, and this is why it has spread now. This is a different duration… So, training includes short, medium, and long-term courses up to two years of field epidemiology training, and the beauty of this program is that it's actually practice-based training — it’s not just classroom. It supported different countries in preparing an epidemiology workforce to tackle various epidemics and health problems, and the program is gaining momentum..."* – KII 5 |
|  | Coordinated public health crisis responses | Refers to the ways public health networks contribute during emergencies or conflicts, including direct intervention, expert mobilization, research, documentation, and advocacy, often responding flexibly to complex regional crises. | Responding to public health crises was discussed by six participants. | *“The war on Gaza, we wrote to the UN Secretary General. We written to some heads of state, in Britain, for example, and some of the Arab countries, we wrote to heads of state. On issues of Gaza, we asked international organisations to support Gaza humanitarian efforts” – KII 16* |
|  | Regional research and knowledge generation | Refers to the collaborative generation and dissemination of knowledge within public health networks, often resulting in regionally relevant publications, reports, and policy documents. | Research was discussed by six participants for networks' contribution to scholarly knowledge and regional evidence generation. | *"So, I mean, there are different things that we’re doing together.. we developed thirteen case studies, which—many of them—are published also. The studies are published in the special edition that we had just last year for evidence and policymaking. And these case studies that we developed jointly together were about… how the countries used local or general evidence—global evidence—for policymaking..."— KII 14* |
|  | Health service delivery contributions | Encompasses notable accomplishments in the delivery and improvement of health services. | Two key informants mentioned health services as a success story. | *"EMPHNET has supported key public health services such as immunization in various countries, enhancing the reach out, the coverage of immunization, and all these things. And this has been really unique, in particular in some countries where problems of access are there."- KII 5* |
|  | Evidence-informed policy impact | Refers to the ways public health- related networks contribute to policy change. | Policy change was discussed by two participants, to illustrate how certain networks extended their work beyond research and knowledge sharing to influence national policies. | *"The work initially started with funding in Syria, Jordan, and Tunisia… They worked on studies, and these studies were aimed at developing tobacco control legislation."* – KII 9 |
|  | National institutionalization | Refers to efforts by regional networks to support, promote, or collaborate with the creation and strengthening of national-level public health or epidemiology associations. | The establishment of national entities was discussed by two participants who reflected on IEA’s role in supporting the creation and engagement of national public health and epidemiological associations. | *"I think some of the most interesting stories is to have promoted the creation of several national entities or national entities… So, the Iranian success is fabulous…the Egyptians have started… the Tunisians are on… they have their own system... the Saudis have created their own epidemic association, but they have not been very cooperative recently. I don't know what the problem is… So that has been going well." –* KII 6 |
| **Theme 4:** Why do regional public health networks struggle or fail? | Funding | Refers to the financial resources required to sustain network activities, with emphasis on the challenges of securing consistent, flexible, and long-term support from donors, institutions, or self-financing mechanisms. | Almost all participants revealed widespread consensus that the lack of a sustained and flexible financing scheme is the most destabilizing challenge facing regional networks | *"Networks are very hard to raise funding for because, for funders, they don't see the direct impact of it. For the members of networks, it's nice because they come together, they meet people, they advance their ideas… But it's hard to say what the concrete outcome of a network is. And it usually—networks—requires a lot of travel funds, so they’re expensive because people have to get together. One thing—you have to get together—and that's expensive… So, I know that over my lifetime, I’ve tried to raise money for networks many times, and it’s difficult. It's much easier to raise funding for a specific research project or a specific activity that has a certain impact."* – KII 7 |
|  | Governance and leadership | This category refers to the informality of networks, over-reliance on individual leaders, lack of institutional ownership, absence of succession planning, leadership transitions, or limited time and capacity among coordinators or steering members. | Eight participants highlighted governance and leadership as a main challenge, with the complex tensions surrounding how networks are structured and led. | *"Really, to run a network well, you need a full-time coordinator… and I was doing it on top of everything else... I was (an administrator) … I was doing research… I was teaching, and I was doing this on the side… So you know, it's really tough to find the time, and I did it because I valued it. And that’s again related to the funding because if you don't have funding for a full-time coordinator, then the person has to squeeze it into their time, and the steering committee, it was the same thing; they were doing this on top of everything else that they do."* |
|  | Political and social context | Refers to the external geopolitical, social, and institutional conditions, such as conflict, inequality, nationalism, or travel restrictions, that impact the functioning, cohesion, and inclusivity of regional public health networks. | The political and social context challenges were raised by nine key informants, highlighting how political fragmentation, structural inequities, and instability hinder regional public health collaboration. | *"also the main thing was that they should talk in Arabic this was easy for social scientists, especially if they were trained in Arabic…But it was a big challenge for the biomedical and health scientists, who usually are trained in English or French…And then they're obliged to speak and present their, you know, abstract or presentations in Arabic" – KII 1* |
|  | Human resource challenges | Refers to the people, both individuals and institutional staff, responsible for sustaining a network’s operations, with emphasis on challenges related to member engagement, lack of dedicated personnel, and dependence on a small group of active contributors. | Seven key informants talked about the challenges of human resources, emphasizing the massive reliance of networks on a small number of highly committed individuals. | *"A second thing is, of course, the non-sustainability of these members... People come and go, and because of the non- formality, you don't have ... something that ties them to the network…. There's nothing binding for members, so as they're available, they support; as they become busy or move on to different places, they are no longer there… So, sustainability is always an issue. This comes with a lack of accountability also..."* – KII 2 |
|  | Lack of institutional continuity | Refers to the pattern of irregular, short-lived initiatives within networks that lack consistent follow-through, long-term planning, or structural support to maintain momentum and lasting engagement. | Ad-hoc activities were highlighted by six key informants who described the struggles networks faced in sustaining long-term momentum. | *”* *Unfortunately, all of those were rather…You know, they would last for maybe a year or few months and then they wither away...”* – KII 3 |
|  | Conflicting individual and institutional interests | Refers to the internal conflicts and power struggles within networks, where individuals or institutions pursue self-interest, such as leadership, influence, or funding, undermining trust, equity, and the shared mission of the network. | Competition within the network was raised by four key informants who described tensions within networks surrounding leadership, institutional dominance, and individual agendas. | *"But what ended it was… it was a network of individuals, not institutions… OK. So that's, I mean, I think one has to kind of think about what kind of network one wants, whether it's a network of individuals or a network of institutions. And I mean, I think what ended up happening is that some of the individuals involved saw this as a way to build up their positions in their institutions… So they saw it as a way of fundraising for their institutions, and that, I think, was the main thing that undermined it—they had their own agendas, basically as individuals, and they were thinking mostly of their own power within their institutions, and that was not what the network was designed to do."* – KII 7 |
|  | Linguistic fragmentation | Refers to the communication challenges within networks arising from the region's multilingual context, where differences in language use, Arabic, English, French, Persian, and others, impact participation, inclusivity, and knowledge sharing across disciplines and countries. | Language was raised by three key informants who discussed how linguistic diversity posed both logistical and conceptual challenges for regional networks. | *"I wouldn't say that linguistically there's a hard and fast barrier because, for example, Iran, you know are usually one of the most assertive countries in anything to do with scientific research and publishing… You know, they're happy to have a forum like this, and obviously they're speaking a different language from basically else in the forum… So that's, you know, it's not all that… You know their first languages, so that in itself isn't a problem… But the fact that we have a region that's not the Middle East and North Africa or that's not the Arab world or whatever, wants to call it, that's a bit of a challenge…"* – KII 4 |
| **Theme 5:** What is needed to sustain regional public health networks? | Governance and leadership arrangements | A set of recommendations focused on building effective and sustainable networks through appropriate institutionalization, including formal structures, secretariat hosting, leadership rotation, and clarified roles and mandates. | Nine participants emphasized the need for institutional, rather than individual, leadership to ensure network sustainability. | *"And then being able to establish a technical secretariat or technical body because, in my opinion, there is no network or anything that can succeed without people who are dedicated. Even if it is a small number of people, if they are dedicated to work in this area, it will succeed. But if people are all part-time and they don't have time and they just come for meetings, then they will meet, they will discuss, and then they will get apart and then forget about things until they come back...."* – KII 5 |
|  | Mechanisms for sustained communication and coordination | Networking Tools are the digital and in-person mechanisms that enable collaboration, communication, and coordination among members of a public health network. | Nine key informants discussed the importance of utilizing networking tools, both digital and in-person mechanisms, to strengthen collaboration and sustainability. | *"And have a regular program—meetings—because, you know… in terms of sustainability, you need to keep people always posted, informed. If you have any news, any update, give feedback, keep them posted… Having regular meetings and regular programs. This can help a lot—linking these kinds of networks to the officials, for example… linking with Ministries of Health, linking between all the national entities… making them closer to each other..."* – KII 14 |
|  | Sustainable and diversified financing | Sustaining Financing refers to establishing reliable, long-term financial models for public health networks, often through mechanisms such as membership fees, institutional support, or mixed funding approaches. | Sustaining financing was raised by nine key informants who discussed the importance of establishing long-term, reliable financial models. | *"There is a lot of philanthropy activity in the region. It tends to be mostly humanitarian, which is perfectly understandable given all the humanitarian needs in the region….But there's very little funding for more long-term kind of… you know organizations like us that have a long-term vision of trying to build an educated…capable…community of researchers and thinkers and writers…”-* KII 15 |
|  | Strategic planning mechanisms | Refers to the process of defining clear goals, vision, and frameworks for public health networks. | Eight participants emphasized the importance of strategic planning by defining clear purpose, structure, and evaluation mechanisms. | *"I think we have lessons to learn, and as much as I talk of EMPHNET now as a success story, if, on the other hand, you ask me about unsuccessful stories, I also have in the region here where, you know, some public health networks were not successful and… The difference, in my opinion, is what we were talking about. So, the difference is in… The orientation having clear, you know, policy direction, having a clear strategy from the start..."* – KII 5 |
|  | Human resource sustainability | Refers to recommendations related to human resources in the networks. | The critical role of human resources in forming and sustaining public health networks was discussed by six key informants | *" Networking is not about setting up a structure… networking is about having people committed" –* KII 10 |
|  | Incentives for sustained engagement | Refer to the various forms of motivation, financial, professional, or relational, that encourage individuals and institutions to actively participate in public health networks. | Incentives were discussed by five participants, who emphasized their importance in motivating both individuals and institutions to participate and remain committed to networks. | *"You can set up prizes. You can. You know, prizes is quite a good thing to do… You can…You know there's ways in which you can, depending on what the point of the network is…You can, you know, you can attract young talent....You can do this. You can do that…You can offer little scholarships…You can do writing"* – KII 4 |
|  | Building trust | Refers to the recommendation of establishing reliability between networks’ members and partners. | Building trust was discussed by two participants highlighting the importance of transparency, openness, and consistent communication. | *"…. and then finally, I think, and this is a bit of a cliche, but to be as open and transparent as possible… to build trust, it's very difficult …. people in this region for very good reasons don't have much trust and you have to earn their trust…. "* – KII 15 |
|  | Understanding the political and social context | Refers to the broader environment in which public health networks operate, including political sensitivities, institutional relationships, regional conflicts, and social dynamics. | Understanding political and social context was mentioned by one participant as public health networks must be fully aware of the political and social environments in which they operate. | *"And understands the politics of the region and the issues in the region and that can advocate and advocate with government, advocate with other organizations…”-* KII 7 |
