# Supplementary material for: What makes public health-related networks work? Qualitative insights from the Eastern Mediterranean and Arab world
Source: Front Public Health. 2026 Aug 3;14:1880808. doi: 10.3389/fpubh.2026.1880808 (PMC13478113; doi:10.3389/fpubh.2026.1880808)
Supplement: Supplementary file 4 [file Table_4.docx]

**What Makes Public Health-related Networks Work? Qualitative Insights from the Eastern Mediterranean and Arab World**

**Ahmad Mandil^1^, Maya Hassan^2^, Iman Nuwayhid^2*^**

^1^Epidemiology Department, High Institute of Public Health, University of Alexandria, Alexandria, Egypt

^2^Faculty of Health Sciences, American University of Beirut, Beirut, Lebanon

Supplementary Material - A glimpse into public health–related networks in the EMR and Arab World

| Public health-related network | Timeline | Membership | Geographical Scope | Mandate | History of the network | Main Milestones |
| --- | --- | --- | --- | --- | --- | --- |
| Reproductive Health Working Group (RHWG) | 1988 – ongoing | Individual-based network | The Arab world and Turkey | - To generate contextual knowledge about women’s reproductive health in the Arab world and Turkey; - To strengthen capacity for rigorous research; - And to inform policies and practices in the region (1-6) | - The RHWG began under the influence of the Middle East Awards (MEAwards), a successful competitive grants programme created by the Cairo office of the Population Council. - The network received continued funding from the Ford Foundation for over 20 years, followed by support from the Canadian International Development Research Council. - From 1988 to 2004, the RHWG was based at the Population Council’s Regional Office for West Asia and North Africa in Cairo, Egypt. - Since 2004, the group has been hosted at the Faculty of Health Sciences at the American University of Beirut (AUB) in Lebanon (3). | - The Giza study, which examined women’s experiences and perceptions of their reproductive bodies, prompted several projects by different network members in various countries, fostering RHWG’s cohesiveness (3). - This collaboration led to the creation of the Choices and Challenges in Changing Childbirth (CCCC) network, which remains affiliated with RHWG and presents research at annual meetings (4). - The theme of violence, conflict, and health developed organically from the historical experience of Palestinian suffering and became linked to RHWG as violence spread throughout the region after events such as the Iraq war, Arab uprisings, and the conflict in Syria. - This theme now involves studies as the one on reproductive health and rights in East Jerusalem, which investigates the effects of biopolitics and militarisation on the experiences of pregnancy and birth among Palestinians (36), illustrating how RHWG research responds to evolving regional troubles (37). |
| The Arab Forum for Social Sciences and Health (AFSSH) | 2002-2018 | Individual-based network | The Arab world | Research production and policy influence | - Ford Foundation–funded forum hosted by the University of Balamand in Lebanon. | - Contributed to the development of anti-smoking legislation in the region (7). - Brought together health scientists alongside social and behavioral scientists across the Arab world, fostering interdisciplinary dialogue. |
| Arab Public Health Association (ArPHA) | 2014-ongoing | Individual-based network | The Arab world | - To strengthen the public health profession through defining standards, strengthening practice and supporting all public health professionals, organisations and all those who contribute to the population’s health from all disciplines in their practice, work and endeavours. | - ArPHA was established in 2014, following discussions among regional public health leaders, which convened experts to explore the creation of a dedicated public health association for the region. | - Issued an official statement addressing the public health consequences of the earthquakes in Turkey and Syria. - Contributed to advocacy on the impact of war and displacement on the wellbeing of women and girls in Gaza. - Organized regional knowledge-exchange initiatives, including the COVID-19 webinar “Exit Strategy: The ‘New’ Normal,” (53). |
| The Eastern Mediterranean Association of Medical Editors (EMAME) | 2004-ongoing | Individual-based network | The EMR | - To promote cooperation and communication between editors of medical journals within the WHO Eastern Mediterranean Region and internationally; - To advance e-healthcare through dissemination of high-quality scientific medicine, improve editorial standards and professionalism in medical editing; - To encourage research in peer review and medical editing; - And to provide continuing education for medical journal editors through regular conferences and courses (9). | - EMAME was established as part of the World Health Organization Eastern Mediterranean Regional Office (EMRO) (8, 42), following the first and second regional conferences on medical journals held in Cairo in 2003 and Riyadh in 2004, respectively (8). - The association cooperates with the World Association of Medical Editors (WAME), the Forum for African Medical Editors (FAME), and other international associations in the field of medical journal publishing. | - Hosted multiple regional workshops on ethics in scientific writing and journal publishing, including:  1. A workshop for more than 25 biomedical journal editors at Iran University of Medical Sciences in Tehran in 2020 (10); 2. A workshop in Lebanon for Ministry of Public Health staff involved in the COVID-19 response (11); 3. Workshops organized in the United Arab Emirates in collaboration with Dubai Health Authority in 2018 (12) and in Ajman in 2015 (13).  - Produced the Manual for Editors of Health Science Journals (14). |
| The Eastern Mediterranean Public Health Network (EMPHNET) | 2009 – ongoing | Institutional-based network | 13 EMR countries | - To prevent and control diseases by strengthening the public health workforce, conducting operational research, enhancing public health programs, and promoting knowledge exchange and networking. | - EMPHNET is a public health nonprofit organization that was established in 2009 to endorse better health in the EMR and beyond. | - Srengthened the public health workforce in the EMR through sustained support for Field Epidemiology Training Programs (FETPs) across the region. - Through the Workforce Capacity (WFC) unit under the CEAE, provided customized training in applied epidemiology, emergency preparedness, immunization, and other public health areas (17). - Supported over 15 countries and implemented more than 35 FETP tiers, resulting in over 200 cohorts and more than 3,000 graduates to date (15). - Contributed to the elimination of vaccine-preventable diseases, including polio (18). - Supported the implementation of the One Health approach in the EMR (19). - Established the Eastern Mediterranean Non-Communicable Disease Research and Prevention Center (NCDsRC) to strengthen NCD prevention efforts (20). - Created EpiShares as a regional platform for health communication and knowledge exchange among public health experts, FETP residents, and graduates (21). - Played a key role in infectious disease control during the COVID-19 pandemic, including case investigation, arrival screening, isolation, case transfer, risk communication, and infection prevention and control training (22). - Operated the Public Health Emergency Management Center (PHEMC) as a hub for information sharing, coordination, and response during epidemics (23). - Advanced maternal and child health initiatives across the region (16). - Promoted the integration of digital health solutions and electronic registries (e-registries) within public health systems. - Sustained operations for over 16 years despite persistent regional challenges, attributed to maintaining a strong regional presence, tailoring interventions to country-specific cultural, social, and political contexts, and investing in local capacity to support long-term sustainability (16, 24, 27,28). |
| The Middle East and North Africa (MENA) chapter of the International Epidemiological Association (IEA) | Ongoing | Individual-based network | EMR | - To facilitate communication among researchers and educators conducting epidemiological studies worldwide; - And to promote epidemiology across various health fields, including social, community, and preventive medicine. | N/A | - Hosting workshops on scientific writing and outbreak investigations. - Hosting collaborative sessions on disease prevention, conflict challenges, and health systems. - Hosting annual scientific workshops for fruitful discussions within the public health community locally and in the region (25, 26). |
| The Eastern Mediterranean Region Academic Institutions Network (EMRAIN) | 2010 – N/A | Institutional-based network | EMR (11 public health institutions) | - To amplify the collective voice of academic public health institutions, enabling them to work together and become respected players in public health and health systems policy reform at the regional level (29). | - EMRAIN was formally launched in March 2010, following a recommendation made at a consultative meeting in 2009. - The increase in public health programs in the EMR region, along with critical gaps in some countries, highlighted the need for collaboration among public health institutions to address these challenges (41). - The initial EMRAIN secretariat was hosted by the Faculty of Health Sciences at AUB, and the network included 11 EMR/Arab World public health institutions and three collaborators. | - During its active years, EMRAIN did not limit its work to public health education. Instead, it created a sustainable platform for dialogue among all partners concerned with public health and health system strengthening, especially policymakers. Its priority areas of work included noncommunicable diseases, complex emergencies, strengthening health systems, emerging infectious diseases, and maternal and child health and nutrition (30) |
| The Arab Council for the Social Sciences (ACSS) | 2008-ongoing | Individual and institutional- based network | The Arab world | - To identify and address the needs of social scientists and their communities, enhance the capacity of researchers and institutions, promote independent and high-quality research, and provide forums for exchange and communication. | - The network emerged following extensive research, consultation, and planning led by a Steering Committee of senior Arab scholars. - From 2006 to 2008, the Committee thoroughly assessed the need for such a body and, through five formal meetings in Beirut, Dubai, Cairo, Amman, and Tunis, respectively (44-52), defined its objectives, functions, and institutional structure. - This preparatory phase, supported by the International Development Research Centre (IDRC) of Canada and the Ford Foundation’s regional office in Cairo (43), culminated in the formal registration of ACSS in 2011, with the aim of strengthening the social sciences in the Arab region. | - ACSS has supported 413 grantees and fellows, including 234 New Generation Fellows, through initiatives such as the Early Career Fellows Program, Paradigms Factory, and the ACSS-FMSH Postdoctoral Mobility Program, as well as a range of research grants and collaborative programs (44-52) - ACSS has organized seven thematic and methodological working groups, including groups focused on violence in the Arab region, critical security studies, ethnography, regionalisms, research on the arts, gendering the archive, and safeguarding knowledge production. - In addition to fellowships and working groups, ACSS has actively advanced initiatives such as the Arab Public Data Initiative, the Critical Humanities Initiative, the InterAsia Partnership, Mapping the Production of Knowledge on Women and Gender, and New Research Agendas on Gender, thereby strengthening cross-program collaboration and impact (43). |
| Network for Evidence and Data to Policy (NEDtP) | 2019-ongoing | Institutional-based network | EMR (22 countries) | - To support evidence-informed national health policy-making and to enhance regional and national technical capacity for utilizing evidence in health policy development. - To collaborate with Member States to enhance the use of evidence in policy development (32). | - NEDtP was established in 2019 by the WHO/EMRO Regional Committee for the Eastern Mediterranean as part of a framework for “improving national institutional capacity for use of evidence in health policy making in the Eastern Mediterranean Region” (31). - The network currently includes 30 institutions from 22 EMR countries in the region. | - NEDtP has produced multiple policy tools, including a policy brief template that guides effective policy brief writing (33), procedural steps and functions for establishing a national programme for guideline adaptation (34), the rapid advice tool for country action on evidence-informed policy-making, which assists countries in quickly translating evidence into policy advice (35), and a glossary defining key terms related to evidence-informed policy-making (36). - Additionally, one of the most significant achievements is developing a guide on evidence, policy, and impact for evidence-informed decision-making (37-40). |

**References:**

1. Giacaman R, Al-Ryami A, Bashour H, DeJong J, Gaballah N, Gherissi A, et al. Importance of research networks: the reproductive health working group, Arab world and Turkey. Lancet. (2014) 383:483–5. doi: 10.1016/S0140-6736(13)62442-3
2. Reproductive Health Working Group Who we are Beirut: RHWG (2025).
3. DeJong J, Zurayk H, Myntti C, Tekçe B, Giacaman R, Bashour H, et al. Health research in a turbulent region: the reproductive health working group. Reprod Health Matters. (2017) 25:4–15. doi: 10.1080/09688080.2017.1379864
4. Kabakian-Khasholian T, El-Kak F, Shayboub R. Birthing in the Arab region: translating research into practice. East Mediterr Health J. (2012) 18:94–9. doi: 10.26719/2012.18.1.94
5. Hamayel L, Hammoudeh D, Welchman L. Reproductive health and rights in East Jerusalem: the effects of militarisation and biopolitics on the experiences of pregnancy and birth of Palestinians living in the Kufr ‘Aqab neighbourhood. Reprod Health Matters. (2017) 25:87–95. doi: 10.1080/09688080.2017.1378064
6. DeJong J, Heidari S. Gendered bodies and reproduction in the Arab countries and Turkey. Reprod Health Matters. (2017) 25:1–3. doi: 10.1080/09688080.2017.1380540
7. Information and Research Center – King Hussein Foundation. Arab Meeting for Social and Health Sciences (AMSHS) [in Arabic]. Amman: IRCKHF.
8. Jawaid SA. Birth of eastern Mediterranean Association of Medical Editors (EMAME). Pak J Med Sci. (2004) 20:1–2.
9. World Health Organization Regional Office for the Eastern Mediterranean. About EMAME. Cairo: World Health Organization Regional Office for the Eastern Mediterranean (2025).
10. World Health Organization Regional Office for the Eastern Mediterranean. Ethics in scientific writing and journal publishing. Cairo: World Health Organization Regional Office for the Eastern Mediterranean (2020).
11. World Health Organization Regional Office for the Eastern Mediterranean. Enhancing scientific Writing and Publishing in Lebanon. Cairo: World Health Organization Regional Office for the Eastern Mediterranean (2022).
12. World Health Organization Regional Office for the Eastern Mediterranean. EMAME–DHA Collaborative Workshop on scientific Writing and Publishing. Cairo: World Health Organization Regional Office for the Eastern Mediterranean (2018).
13. World Health Organization Regional Office for the Eastern Mediterranean. Second International Congress on Medical Writing. Cairo: World Health Organization Regional Office for the Eastern Mediterranean (2015).
14. World Health Organization Regional Office for the Eastern Mediterranean. Manual for editors of Health Science Journals. Cairo: World Health Organization Regional Office for the Eastern Mediterranean (2009).
15. Khader Y, Shalabi D, Daoud L, Al Bakri D, AlHamawi R, Saad RK, et al. The role of public health networks in strengthening public health systems: the case of EMPHNET in the eastern Mediterranean region. Front Public Health. (2025) 13:1641842. doi: 10.3389/fpubh.2025.1641842
16. Al Nsour M, Kaiser R. Networking for applied field epidemiology: eastern Mediterranean public health network (EMPHNET) conference 2011. East Mediterr Health J. (2011) 17:984–8.
17. Amiri M, Al Nsour M, Alonso-Garbayo A, Al Serouri A, Maiteh A, Badr E. Health system resilience in the eastern Mediterranean region: perspective on the recent lessons learned. Interact J Med Res. (2022) 11:e41144. doi: 10.2196/41144
18. Al Nsour M, Bashier H, Al Serouri A, Malik E, Khader Y, Saeed K, et al. The role of the Global Health development/eastern Mediterranean public health network and the eastern Mediterranean field epidemiology training programs in preparedness for COVID-19. JMIR Public Health Surveill. (2020) 6:e18503. doi: 10.2196/18503
19. Al Gunaid M, Lami F, Jarour N. A collaborative initiative to strengthen sustainable public health capacity for polio eradication and routine immunization activities in the Eastern Mediterranean Region. JMIR Public Health Surveill. (2019) 5:e14664. doi: 10.2196/14664
20. Hailat E, Amiri M, Debnath NC, Rahman M, Islam MN, Fatima Z, et al. Strengthening the one health approach in the eastern Mediterranean region. Interact J Med Res. (2023) 12:e41190. doi: 10.2196/41190EMPHNET
21. Eastern Mediterranean Public Health Network. EMPHNET. Amman: EMPHNET (2019). Available online at: https://emphnet.net/ (Accessed August 28, 2025).
22. Noormal B, Eltayeb E, Al Nsour M, Mohsni E, Khader Y, Salter M, et al. Innovative approaches to improve public health practice in the Eastern Mediterranean Region: findings from the sixth Eastern Mediterranean Public Health Network regional conference. JMIR Public Health Surveill. (2019) 5:e11382. doi: 10.2196/11382
23. Eastern Mediterranean Public Health Network. The way forward for pertussis control in the Middle East and North Africa: focus on maternal immunization. Amman: EMPHNET. Available online at: https://emphnet.net/media/3ycjj3ut/sanofi_y23_pertussiscontrol_project-breif.pdf (Accessed January 9, 2025).
24. Eastern Mediterranean Public Health Network. Public Health Emergency Management. Amman: EMPHNET (2019).
25. International Epidemiological Association. About IEA. International epidemiological association. Available online at: https://ieaweb.org/ (Accessed November 19, 2025).
26. Mandil A, Chaaya M, Saab D. Health status, epidemiological profile and prospects: eastern Mediterranean region. Int J Epidemiol. (2013) 42:616–26. doi: 10.1093/ije/dyt026
27. Eastern Mediterranean Public Health Network. Toward public health resilience in the eastern Mediterranean region: findings from the seventh eastern Mediterranean public health network regional conference. Interact J Med Res. (2023) 12:e36356. doi: 10.2196/36356
28. Al Nsour M. The eastern Mediterranean public health network: a resource for improving public health in the eastern Mediterranean region. JMIR Public Health Surveill. (2019) 5:e14992. doi: 10.2196/14992
29. World Health Organization Regional Office for the Eastern Mediterranean. Consultative Meeting on Development of a public Health Leadership Programme and Strengthening the Network of academic Institutions in the Eastern Mediterranean Region. Cairo: World Health Organization Regional Office for the Eastern Mediterranean (2013).
30. World Health Organization Regional Office for the Eastern Mediterranean. Side Session of public Health Institutes from the Eastern Mediterranean Region, Marrakech. Cairo: World Health Organization Regional Office for the Eastern Mediterranean (2014).
31. World Health Organization Regional Office for the Eastern Mediterranean. First Meeting of regional Network of Institutions for Evidence and data to Policy. Cairo: World Health Organization Regional Office for the Eastern Mediterranean (2021).
32. World Health Organization Regional Office for the Eastern Mediterranean. Scope of and Strategies for the Network of Institutions for Evidence and Data to Policy (NEDtP) in the Eastern Mediterranean Region. Cairo: World Health Organization Regional Office for the Eastern Mediterranean (2025).
33. Lavis JN, Kheirandish M, Al-Ansary L, Basaleem H, Hajjeh R, Al-Mandhari A, et al. Next generation support and stronger institutions for evidence-informed policy-making. East Mediterr Health J. (2023) 29:495–7. doi: 10.26719/2023.29.7.495
34. World Health Organization. Policy Brief Template: how to Write an Effective Policy Brief. Geneva: World Health Organization (2024).
35. World Health Organization. Establishing a national Programme for Guideline Adaptation: key Steps and Functions. Geneva: World Health Organization (2024).
36. World Health Organization Regional Office for the Eastern Mediterranean. Rapid Advice tool for Country action on Evidence-Informed Policy-Making. Cairo: World Health Organization Regional Office for the Eastern Mediterranean (2019).
37. World Health Organization. Evidence-Informed Policy-Making: a Glossary of key Terms. Geneva: World Health Organization (2025).
38. World Health Organization. Evidence, Policy, impact: WHO guide for Evidence-Informed Decision-Making. Geneva: World Health Organization (2022).
39. World Health Organization Regional Office for the Eastern Mediterranean. Intercountry Consultative Meeting for the Network of Institutions for Evidence and Data to Policy (NEDtP) and Finalizing the regional action plan for Evidence-Informed Policy-Making. Cairo: World Health Organization Regional Office for the Eastern Mediterranean (2020).
40. World Health Organization Regional Office for the Eastern Mediterranean. Developing national institutional capacity for evidence-informed policy-making for health. East Mediterr Health J. (2021) 27:314–5. doi: 10.26719/2021.27.3.314
41. El-Jardali F, Jamal D, Ataya N, Jaafar M, Raouf S, Matta C, et al. Health policy and systems research in twelve eastern Mediterranean countries: a stocktaking of production and gaps (2000–2008). Health Res Policy Syst. (2011) 9:39. doi: 10.1186/1478-4505-9-39
42. Gezairy HA. "Message from the regional director". In: Proceedings of the Eastern Mediterranean Medical Journals Meeting. Cairo: World Health Organization Regional Office for the Eastern Mediterranean (2025)
43. Arab Council for the Social Sciences. History of the ACSS. Beirut: Arab Council for the Social Sciences (2015).
44. Arab Council for the Social Sciences. Foreign Association Establishment Decree. Beirut: Arab Council for the Social Sciences (2008).
45. Arab Council for the Social Sciences. Introduction to the Arab Council for the Social Sciences. Beirut: Arab Council for the Social Sciences (2011).
46. Arab Council for the Social Sciences. Terms of Engagement and Operations Template. Beirut: Arab Council for the Social Sciences (2023).
47. Arab Council for the Social Sciences. Amended Bylaws. Beirut: Arab Council for the Social Sciences (2025).
48. Arab Council for the Social Sciences. Amman Meeting Agenda, March 1–2, 2008. Beirut: Arab Council for the Social Sciences (2008).
49. Arab Council for the Social Sciences. Tunisia Meeting Agenda and Participants list, July 8–9, 2008. Beirut: Arab Council for the Social Sciences (2008).
50. Arab Council for the Social Sciences. Cairo Proposed Agenda, February 7–11, 2011. Beirut: Arab Council for the Social Sciences (2011).
51. Arab Council for the Social Sciences. Beirut workshop summary report, April 6, 2023. Beirut: Arab Council for the Social Sciences (2023).
52. Arab Council for the Social Sciences. Dubai meeting summary report, November 6, (2023). Beirut: Arab Council for the Social Sciences
53. Arab Public Health Association. About the Arab Public Health Association. Beirut: ArPHA.
